# Formal Comment: Romer study fails at following core principles of reanalysis

**DOI:** 10.1371/journal.pone.0237184

**Published:** 2020-11-18

**Authors:** Jeffrey A. Bridge, Joel B. Greenhouse, Kelly J. Kelleher, John V. Campo

**Affiliations:** 1 Abigail Wexner Research Institute at Nationwide Children's Hospital, Columbus, OH, United States of America; 2 The Ohio State University College of Medicine, Columbus, OH, United States of America; 3 Carnegie Mellon University, Pittsburgh, PA, United States of America; 4 West Virginia University, Morgantown, WV, United States of America; University of New South Wales, AUSTRALIA

Dr. Romer [1] challenges our previously published finding of a significant association between the release of the Netflix series *13 Reasons Why* (*13RW*) and an increase in suicide rates among 10- to 17-year-olds in the United States [2]. Romer attempts to justify his effort at reanalysis by insisting that (1) contagion must be stronger for girls than boys because the lead character is an adolescent girl and (2) our analysis did not consider secular trends in suicide [1].

Regarding his first point, males typically choose more lethal methods such as firearms and account for most of the suicide deaths in this age group [3]. Not only might adolescent boys identify with Hannah Baker’s distress and predicament, but Romer’s contention that “there would not be reason to expect much of a Werther effect” [1] in the case of young males related to *13RW* overlooks the suicide attempt of an adolescent male in the series in the wake of Hannah Baker’s death. This less prominent suicide attempt in the series provides a gender-congruent model of suicide for adolescent males.

With regard to his second point, Romer’s statement that our analysis “did not take into account strong secular trends in suicide” [1] fails to recognize that our analytic approach included two different modeling approaches: (1) an interrupted time series segmented regression that accounted for both the underlying secular trend and seasonal variation in youth suicide rates and (2) the Holt-Winters’ forecasting method, which uses a triple exponential smoothing model to fit and to forecast monthly suicide rate data: one equation for level, one for secular trend, and one for seasonality. A strength of the Holt-Winters method is that it simultaneously accounts for the effects of trend and seasonality [4]. The auto-regression approach with which Romer chose to model these data ignores the well-known seasonal variation in suicide rates and important components of variation in these data that were incorporated in our approach [2]. As a result, his forecasts will have less precision than ours.

Regarding Romer’s reported results, it must be made clear that his analyses in fact *replicate* our main study findings of a significant increase in the suicide rate for boys in the month following the release of *13RW*, as well as no significant association between the series’ release and suicide in girls. Despite his conclusion that “it is difficult to attribute harmful effects of the show using aggregate rates of monthly suicide rates”, his own results section acknowledges that for boys “the increase that Bridge et al. observed in April was replicated” [1] and notes a statistically significant effect size (B = .173, 95% CI = .011 to .335). Furthermore, with respect to girls, Romer states “We found a positive but, in this case **non-significant** increase in April…” (emphasis ours). Rather than clearly stating the statistically significant finding with respect to boys in the abstract, Romer focuses instead on how a non-significant association of the series’ release on suicide rates in girls should somehow be interpreted as the series producing both protective and harmful effects.

A potentially more significant error is that Romer’s time-series analyses neglect to account for the fact that Netflix was actively broadcasting advertisements and series’ trailers throughout March of 2017 that targeted youth and encouraged them to watch this dramatization of an adolescent girl’s suicide. It was for that reason that we had developed a pre-specified time series analysis protocol to manage the gap between promotion and release of the series [2]. Similarly, Romer’s tendency to minimize the observed increase in suicide in April 2017 fails to consider the potential impact of “binge watching” of the series in its first month of release, also discussed in our paper [2].

Perhaps the most cogent challenge to Romer’s reanalysis is provided by Christakis and Zimmerman [5] in an elegantly written JAMA Viewpoint entitled “Rethinking Reanalysis”. The authors put forward core principles of study reanalysis that suggest that the Romer effort may be wanting in many respects, including the presumption of bias. Specifically, Romer appears to be intrigued by the possibility that *13RW* might be associated with a “Papageno effect,” conceptualized as any suicide-protective impact of media reporting or portrayals [6] (“…there is reason to believe that such an effect might be muted by the presence of another phenomenon known as the Papageno effect…”). Despite his speculation that such an effect exists within the available data, there is no statistically meaningful evidence from Season 1 to support a Papageno effect associated with *13RW*. Consequently, his suggestion that the interest of the producers of *13RW* in “portraying the harmful effects of youth culture, especially on young women, may have had some benefits” [1] could be interpreted as gratuitous.

The Romer study fails at following other core principles of reanalysis [5], including (1) not recording the proposed methodological approach in a prespecified protocol at a clearing site such as the Open Science Framework (OSF) preregistration portal (https://osf.io/prereg/) to guard against the reanalysis being interpreted as a “statistical fishing expedition” [5], and (2) failure to articulate how a “straightforward auto-regression analysis” [1] represents a recognizable and significant methodological improvement over our approach [2]. Romer [1] also fails to articulate clearly that ours was one of two independently researched papers [2, 7] published in peer-reviewed journals that found an increase in youth suicide rates associated with the release of *13RW*. In the Niederkrotenthaler et al. [7] paper, the authors extended the pediatric age range to 19 years and found a significant association between the release of *13RW* and suicide in both boys and girls.

In conclusion, our original analysis controlled for the secular trends that Romer mentions and was the product of interdisciplinary team science. Beyond challenging the results of not one, but two independent analyses published just a month apart, Romer fails to meet the basic standards for reanalysis outlined by Christakis and Zimmerman [5]. He highlights null findings to support his published views and dismisses significant associations in his own analysis that run counter to his presuppositions [1]. A well-done reanalysis has potential to advance scientific debate and promote public health [5]. However, a reanalysis lacking adherence to core principles to advance strongly held preconceptions represents, as Christakis and Zimmerman write "… not better science but scientific cacophony [5]."
